# The role of macrophage scavenger receptor 1 (MSR1) in inflammatory disorders and cancer

**DOI:** 10.3389/fimmu.2022.1012002

**Published:** 2022-10-17

**Authors:** Jack Gudgeon, José Luis Marín-Rubio, Matthias Trost

**Affiliations:** Laboratory for Biological Mass Spectrometry, Biosciences Institute, Newcastle University, Newcastle-upon-Tyne, United Kingdom

**Keywords:** MSR1, CD204, macrophages, immunology, cancer, inflammation

## Abstract

Macrophage scavenger receptor 1 (MSR1), also named CD204, holds key inflammatory roles in multiple pathophysiologic processes. Present primarily on the surface of various types of macrophage, this receptor variably affects processes such as atherosclerosis, innate and adaptive immunity, lung and liver disease, and more recently, cancer. As highlighted throughout this review, the role of MSR1 is often dichotomous, being either host protective or detrimental to the pathogenesis of disease. We will discuss the role of MSR1 in health and disease with a focus on the molecular mechanisms influencing *MSR1* expression, how altered expression affects disease process and macrophage function, the limited cell signalling pathways discovered thus far, the emerging role of MSR1 in tumour associated macrophages as well as the therapeutic potential of targeting MSR1.

## Introduction

The innate immune response and the inflammatory response constitute the first mechanisms of host defence. Here, macrophages are crucial innate immune cells that play an essential role by maintaining tissue homeostasis and eliminating pathogens by phagocytosis, the uptake of particulate material (1) Macrophages are found in various tissues and display phenotypic heterogeneity depending on their tissue environment and activation state. The typical nomenclature used to describe macrophage activation splits macrophage populations into two broad states, M1 (classically activated) or M2 (alternatively activated). These states are defined by inflammatory status. M1 macrophages are pro-inflammatory, specialising in pathogen killing whilst M2 macrophages are anti-inflammatory and are responsible for tissue repair and the promotion of cell proliferation (2, 3). This approach to defining macrophages is, however, reductionist and often leads to confusion. It is therefore important to recognise that macrophages exist on a spectrum of activation states, rather than in a binary system (3). Macrophage scavenger receptor 1 (MSR1) has been shown to be important for M2 polarisation (4). Therefore, the role it carries or MSR1 is in part determined by the type of macrophage in which MSR1 is expressed and location within which the macrophages reside.

MSR1, also known as scavenger receptor-A (SR-A) or cluster of differentiation 204 (CD204), was first described in 1979 by Brown and Goldstein (5). They demonstrated that MSR1 mediated the uptake and degradation of acetylated low-density lipoprotein (acetyl-LDL) but not non-modified low-density lipoprotein (LDL). This led to increased intracellular cholesterol deposition, and they postulated that the receptor is responsible for similar effects observed in familial hypercholesterolemia. Further research identified pathologic similarities between lipid-laden cells and foam cells found in the tissue of hyperlipidaemic patients (6). Therefore, the first role described for MSR1 was in the pathogenesis of atherosclerosis, which kick-started further investigations into MSR1. Whilst MSR1 has been shown to be active across the disease spectrum, our understanding of the molecular mechanisms behind its various functions has been marred by incomplete signalling pathways and contradictory experimental results. This therefore introduces a complex view of the role MSR1 plays in macrophage-mediated inflammatory disorders.

In this review we will outline the genetic, epigenetic, and post-translational alterations affecting MSR1, as well as the molecular mechanisms and cell signalling of MSR1. Finally, our interest is to collate the evidence available regarding the clinical implications of MSR1 alterations in different pathologies of the immune system and its role in tumour-associated macrophages (TAMs) in cancer, especially as it has been proposed as a potential biomarker with prognostic value. Finally, we will provide an overview of future avenues for therapeutic targeting of MSR1.

## MSR1 structure and ligand recognition

**Figure 1:**
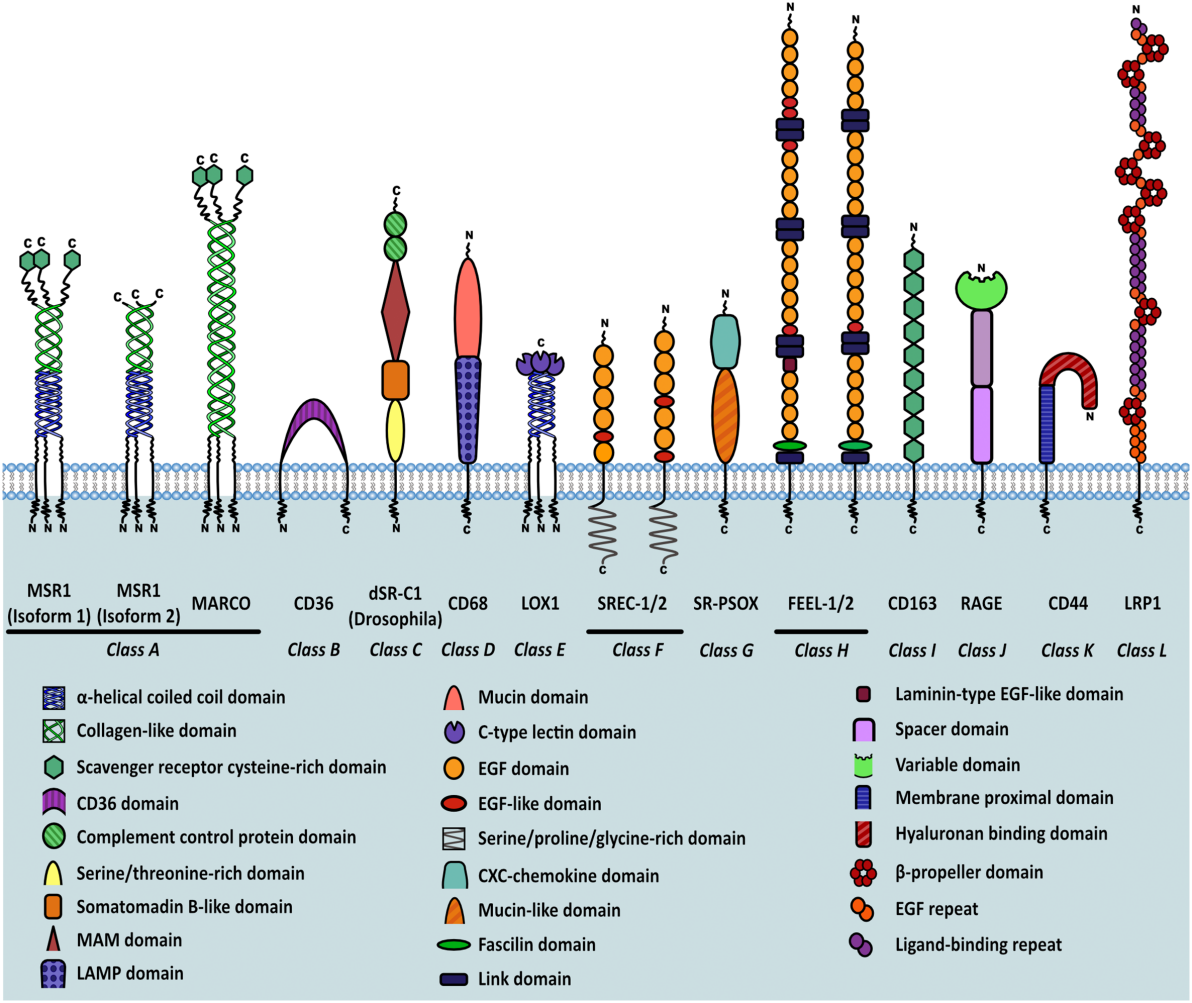
Classification of scavenger receptors and MSR1 isoforms. Schematic representation of different classes of scavenger receptors. Individual domains are identified in the key within the figure.

As more scavenger receptors with similar broad binding specificities were discovered, they were first subdivided into “classes”, based on their primary sequences, and then further subdivided into “types” as a result of sequence variation caused by alternative splicing. This subdivision gave rise to the scavenger receptor family which currently consists of twelve different classes (classes A-L), grouped together by shared functional and ligand binding properties rather than sequence homology (7–9) (**Figure 1**).

The class A scavenger receptors, including MSR1, share a similar ligand repertoire due to the presence of a collagen-like binding domain. These receptors are expressed in a range of different organs but work mainly to clear bacteria, bind and degrade modified lipids, and in the case of SR-A3, protect against reactive oxygen species. Class B scavengers bind high density lipoprotein and influence the development of atherosclerosis. SR-B1 has a protective effect whilst CD36, which also binds oxLDL, has been shown to potentiate atherosclerosis. Expression of class C scavenger receptors is restricted to *Drosophila melanogaster.* CD68, a member of class D, contains a lysosome-associated membrane glycoprotein (LAMP) domain and functions mainly to scavenge oxLDL. The class E scavenger receptors, characterised by presence of a C-type lectin-like domain, function primarily to recognise and remove pathogen associated molecular patterns (PAMPs). Class F scavenger receptors, including SREC-1, bind fungal pathogens, heat shock proteins (HSPs), and apoptotic cells. SR-PSOX belongs to class G and binds oxLDL, phosphatidylserine and mediates phagocytosis of bacteria. It is also the only protein known to share both scavenger receptor and chemokine activities. The class H receptors, FEEL-1 and FEEL-2, are phagocytic receptors that clear apoptotic cells and also mediate angiogenesis. Class I receptor CD163 is similar to other scavenger receptors in its ability to bind gram-positive and negative bacteria but is unique in its haematological role aiding clearance of plasma haemoglobin. RAGE, a member of class J, binds multiple ligands and is classed as a pattern recognition receptor during chronic inflammation or infection. RAGE signalling mediates oxidative stress, inflammation and apoptosis. The class K scavenger receptor CD44 is an endocytic receptor that binds hyaluronan and other components of the extracellular matrix. Finally, LRP1 is a low density lipoprotein receptor that functions primarily to clear plasma cholesterol (10). As is evident, scavenger receptor classes are highly similar in their ligand binding capabilities and function, with multiple classes able to bind and clear pathogens, PAMPS and modified self-molecules. Therefore the scavenger receptor family plays a significant role in host defence.

The *MSR1* gene consists of 11 exons and encodes the class A macrophage scavenger receptors (SR-A); three different isoforms are generated by alternative splicing: SR-AI, SR-AII and SR-AIII. SR-AI and SR-AII are the two predominant isoforms in mammals, the only difference between the two being the lack of a cysteine-rich C-terminus region in SR-AII (11, 12). SR-AIII is a truncated, non-functional variant that has altered intracellular processing and becomes trapped within the endoplasmic reticulum. Presence of this isoform can, however, reduce modified LDL uptake by SR-AI and SR-AII, suggesting a function as a dominant negative isoform as well as a mechanism for regulation of scavenger receptor activity in macrophages (13).

**Figure 2:**
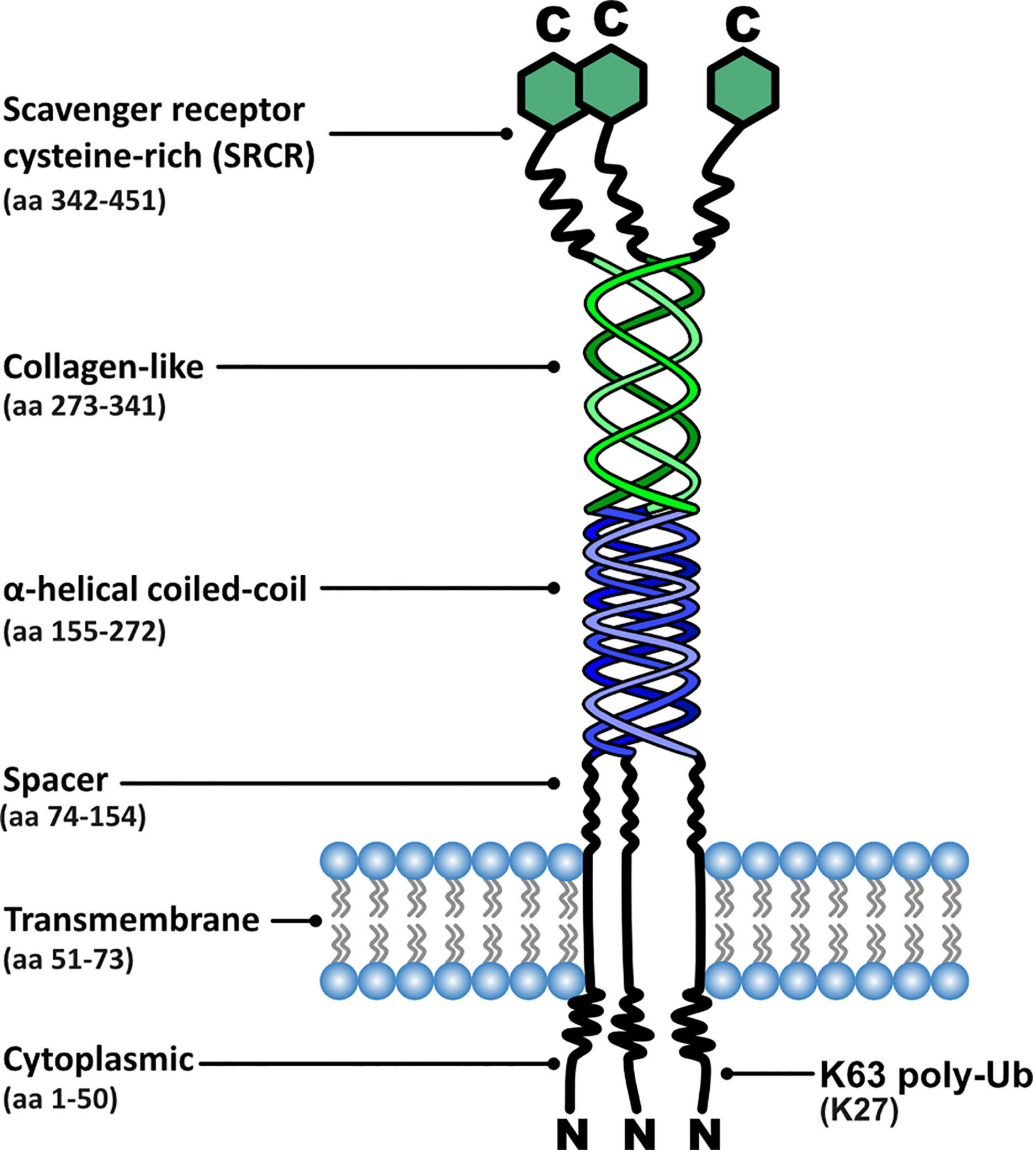
Functional domain organisation of MSR1, including corresponding amino acid (aa) positions and post-translational modification sites relevant to signalling.

MSR1 is a homo-trimeric transmembrane glycoprotein consisting of six distinct domains, with the collagen-like domain and the scavenger receptor cysteine-rich (SRCR) domain being most relevant to its function (14) (**Figure 2**). The site for ligand interaction is found in the extracellular collagen-like domain. Lysine clusters within this domain form positively charged grooves allowing interaction with polyanionic ligands (15). The existence of binding sites within the collagen-like domain explains why SR-AI and SR-AII have identical binding capabilities despite the C-terminal differences.

The ligand repertoire of MSR1 is an unusually diverse array of both endogenous and exogenous ligands (**Table 1**), thus leading to its description as ‘molecular flypaper’ (45). Interestingly, whilst all known ligands share the common feature of being polyanionic, not all polyanions are ligands. This suggests that whilst the possession of multiple negative charges is an absolute requirement, ligand structure also contributes to binding ability (46). Therefore, MSR1 behaves in some way as a pattern recognition receptor.

**Table 1:** List of the endogenous and exogenous ligands known to bind to MSR1 and their effects.

Endogenous ligand	Effects of ligand binding
Acetyl-LDL	Activation, inflammation, and pathogenesis of atherosclerosis (5, 16)
Advanced glycation and product modified proteins	Clearance and pathogenesis of atherosclerosis (17)
Apolipoproteins A-I (ApoAI) and E (ApoE)	Macrophage adhesion (18) Pathogenesis of atherosclerosis and Alzheimer’s disease (18, 19)
Apoptotic cells	Clearance and reduction in inflammation (20)
β-amyloid	Microglia activation and inflammation (21) Protection against Alzheimer’s (22)
Calciprotein particles	Clearance and protection against atherosclerosis (23)
Collagen	Macrophage adhesion (24)
Heat shock proteins (HSP)	Protection against atherosclerosis (25–27)
Maleylated LDL, HDL & albumin	Release of proteases (16)
Oxidised-LDL	Cholesterol deposition and pathogenesis of atherosclerosis (28) JNK-mediated inflammation (29)
Proteoglycans	Macrophage adhesion to extracellular matrix, and pathogenesis of atherosclerosis (30)
**Exogenous ligand**	**Effects of ligand binding**
Berberine alkaloids	Inhibition of LPS endocytosis (31)
Carrageenan	Used as a competitive inhibitor (16, 32)
Crocidolite asbestos	Possible link to asbestosis and mesothelioma (33)
Dextran sulphate	Used as a competitive inhibitor (16)
Double stranded RNA (dsRNA)	Sensing of, and protection against viruses (34–36)
Fucoidan	Used as a competitive inhibitor (16) JNK-mediated inflammation (29)
β-Glucans	Recognition of fungi and bacteria (37)
Human cytomegalovirus (HCMV)	Clearance and protection (38)
Lipopolysaccharide (LPS)	Clearance and protection against bacteria (39)
Lipoteichoic acid (LTA)	Clearance and protection against bacteria (40)
Muramyl dipeptide	NOD2-mediated inflammatory response (41)
Organic dust extract	Regulation of respiratory inflammation (42)
Polyguanylic (poly (G)) and Polyinosinic acid (poly(I))	Used as a competitive inhibitor (16)
Polyinosinic: polycytidylic acid (poly (I:C))	Used as a competitive inhibitor (16)
Silica	*In vitro –* silica induced apoptosis (43) *In vivo –* Appropriate regulation of immune response to silicosis (44)

An interesting binding phenomenon also occurs within the collagen-like domain, referred to as nonreciprocal cross-competition of ligands. In simpler terms, if two different ligands are present, ligand A may be able to completely out-compete ligand B, but ligand B is unable to completely displace ligand A, even at significantly higher concentrations. When comparing the binding dynamics of oxidised-LDL and acetyl-LDL, this property becomes apparent. This has been attributed to the presence of two distinct but overlapping binding sites. Nonreciprocal cross-competition has interesting implications when considering the complex signalling surrounding MSR1 as the receptor may be unable to bind a high affinity ligand due to the pre-existing presence of a lower affinity ligand. Therefore, the order in which MSR1 binds its ligands may have an impact on signalling (47). However, this has yet to be experimentally demonstrated.

Interestingly, despite the receptor being associated with a diverse range of physiological and pathological processes, the cytoplasmic domain lacks a discernible signalling motif. Therefore, to successfully propagate an intracellular signal, MSR1 must associate with other membrane receptors, cytoplasmic components, or rely on post-translational modifications (PTMs) to enable the recruitment of signalling complexes (29, 48).

## Genetic and epigenetic alterations affecting MSR1

**Figure 3:**
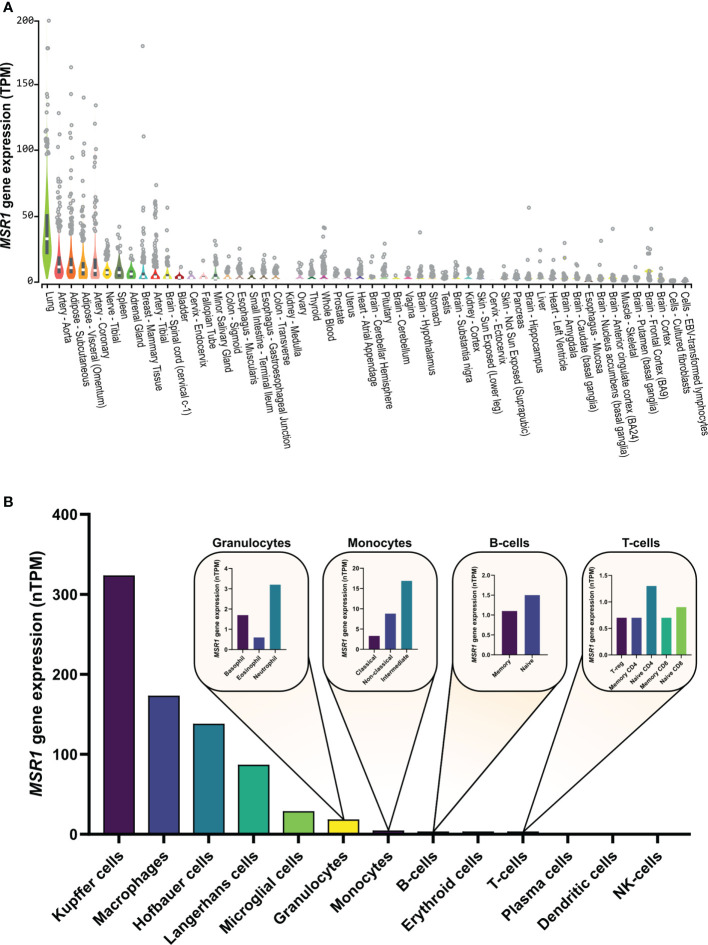
*MSR1* expression in **(A)** normal human tissues and **(B)** human immune cells. Human tissue expression measured by RNA sequencing abundance of *MSR1* transcripts, using a normalization method to calculate transcripts per million (TPM), determined by the GTEx Project (49). Human immune cell *MSR1* expression measured by RNAseq as part of the human protein (HPA) database (proteinatlas.org) (50).

MSR1 is highly expressed in lung, arteries, and adipose tissue (**Figure 3A**). This receptor can be also found at low levels in other cell types: vascular smooth muscle cells (51, 52), astrocytes (53, 54), murine embryonic fibroblasts (35), human lung epithelial cells (36), and a range of endothelial cells (48, 55–57). MSR1 is, as its name suggests, predominantly expressed in various macrophage cell types (**Figure 3B**) (5, 6) and is mainly altered in macrophage-associated physiological and pathological processes including atherosclerosis, Alzheimer’s disease, host defence, and cancer. However, the mechanisms responsible for the alteration of MSR1 expression are not well-studied. Next, we will explore the different mechanisms responsible for changes in MSR1 expression.

## Chromosome 8p deletion

**Figure 4:**
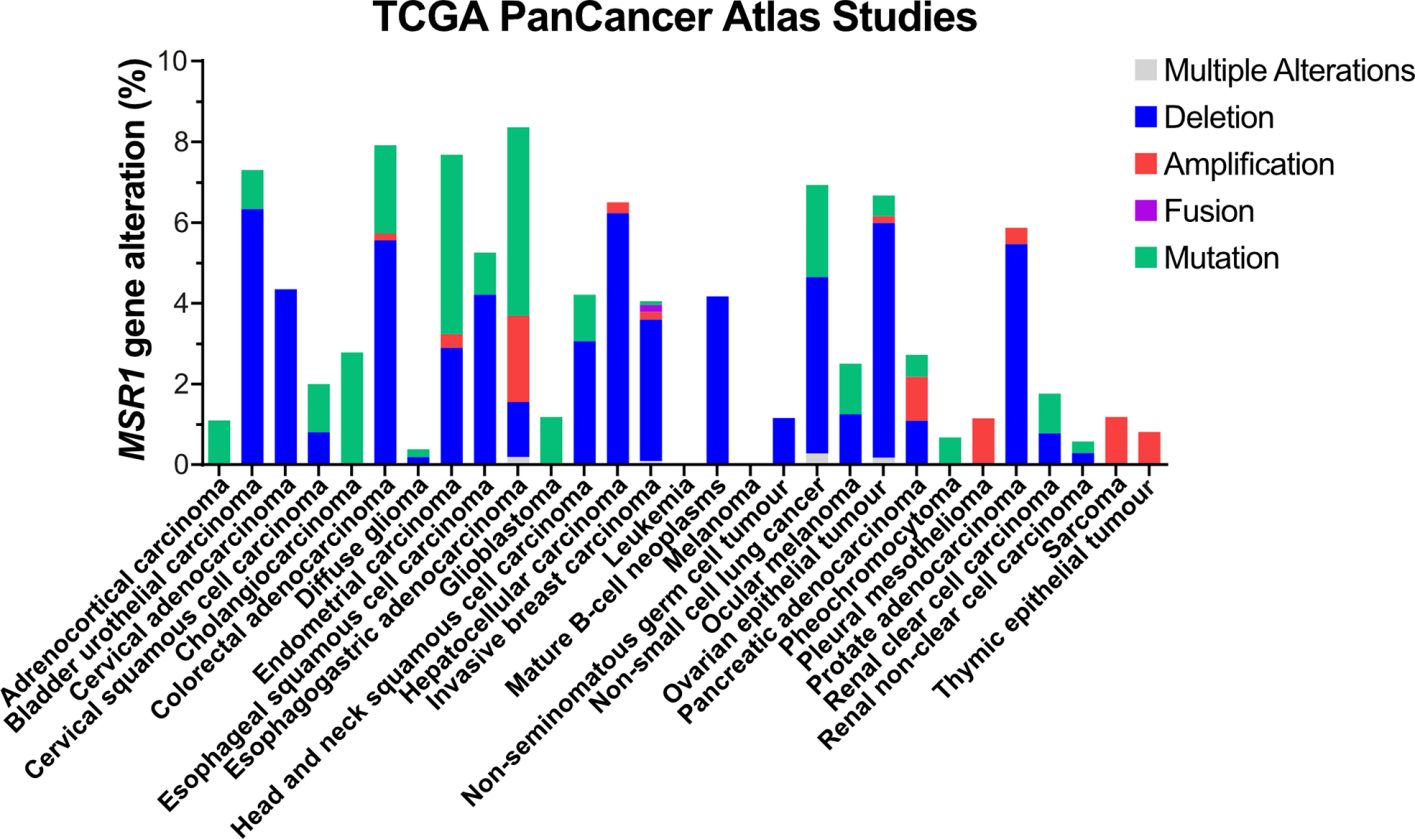
Genetic alterations of MSR1 in human tumour tissues. Data obtained from TCGA PanCancer Atlas Studies in 10967 tumour samples from various origins.

A major event leading to under-expression of *MSR1* is DNA deletion (**Figure 4**). The *MSR1* gene is located on chromosome 8 in humans. The 8p arm is a 6.4 Mb-region that contains 20 other genes, besides *MSR1*, with high frequent loss of heterozygosity (LOH) and homozygous deletion (58). Deletion of this region has been frequently found in different types of human cancers and it is associated with disease progression and poor prognosis. Frequent gene deletions in this region are prognostic markers of cancer such as *HCRP1* (59) and *DLC1* (60) in hepatocellular carcinoma, or *TUSC3* in oral squamous cell carcinoma (61). Also, deletion of the microRNA miR-383 at 8p22 region leads to tumour initiation and prostate cancer metastasis (62). Deletions in this region are also linked with high aggressiveness and risk of disease progression of prostate cancer, even after a radical prostatectomy (63). Insertions into *MSR1* gene have also been observed in gastric adenocarcinoma (COSMIC ID: 8185658). As *MSR1* expression is already low in these cell types, it is unclear how *MSR1* deletion contributes to the progression of cancer. The changes in prognosis seen as a result of deletions in this region are therefore more likely due to loss of other genes in this region.

## MSR1 mutations

Mutations in MSR1 are the second most frequent genetic alteration, which represent 3.3% (1250 out of 38170 tumour samples) according to the COSMIC (the Catalogue Of Somatic Mutations In Cancer) database. In the TCGA PanCancer Atlas Studies, a total of 220 different somatic mutations were identified, of which 35 affected the protein sequence and 3 involved MSR1 in fusion proteins (**Figure 4**). However, MSR1 was not catalogued as a cancer driver by the IntOGen-mutations platform (64). To date, 30,163 variants have been described in the Ensembl database. However, only 3 of them have been attributed to pathogenesis and clinical consequence, NM_138715.3 (MSR1): c.520G>T (p.Asp174Tyr) and c.877C>T (p.Arg293Ter) in prostate cancer (65); and c.760C>G (p.Leu254Val) specifically related to Barrett oesophagus and oesophageal adenocarcinoma (66).

## Transcriptional regulation of MSR1

**Figure 5:**
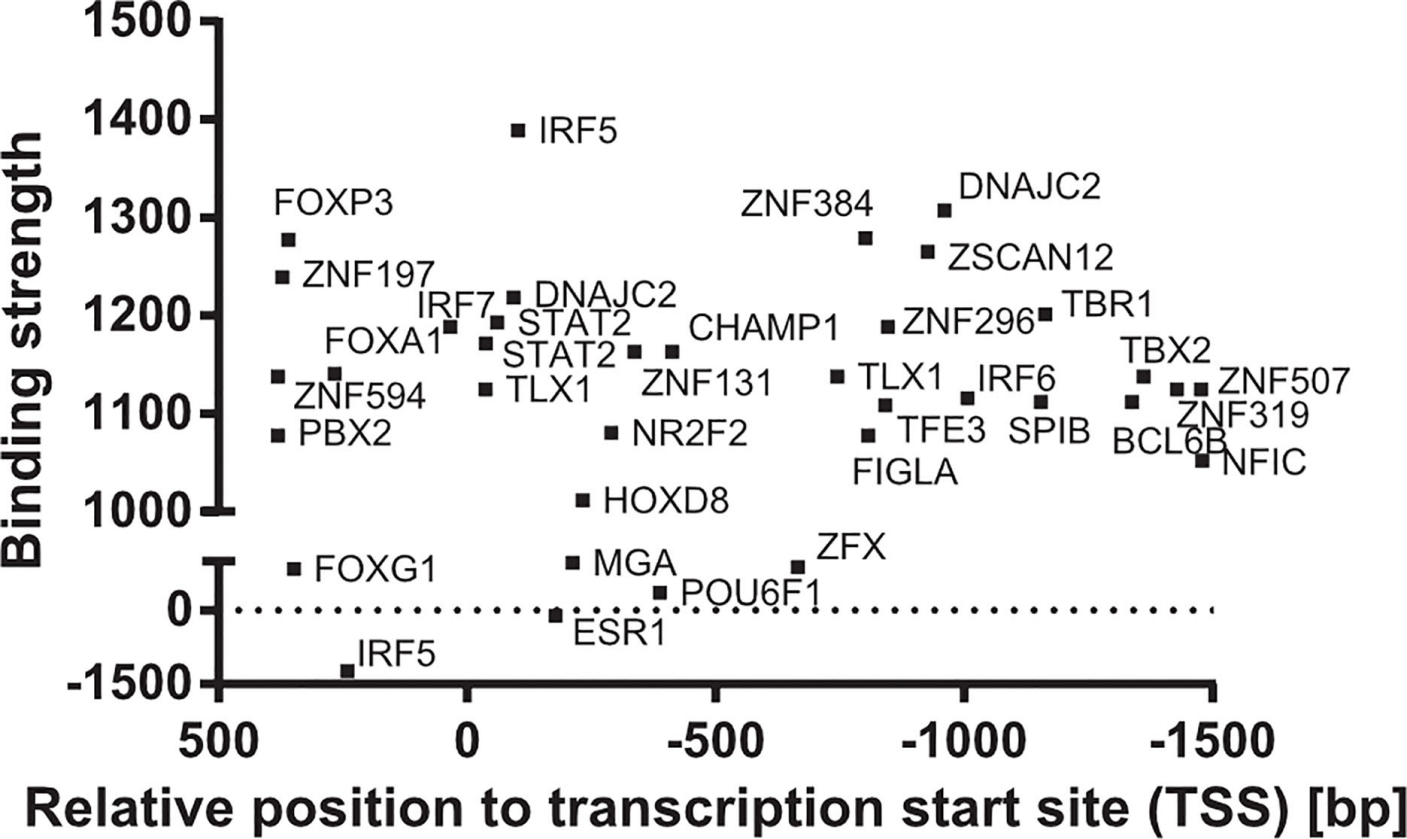
Transcription factors of *MSR1* promoter. Binding sites of transcription factors in the promoter of *MSR1* from experimental and theoretical sources from the TF2DNA database (70).

MSR1 transcriptional expression is regulated by different transcription factors, such as: SPI1, ETS2, c-JUN, c-FOX, and CEBPB (67–69); and predicted transcription factors (70), such as: STAT2, IRF5 and DNAJC2, which are shown in **Figure 5**. The activity of c-Jun and STAT2 in the regulation of MSR1 is interesting as the JAK/STAT and JNK/SAPK pathways are also involved in leptin-induced chemotaxis of monocytes, macrophages, and cancer cells (71, 72). This link may be important for the activity of MSR1 in the tumour microenvironment, as will be discussed later in this review. On the other hand, there are pro-inflammatory cytokines that inhibit MSR1 transcription or reduce MSR1 activity in macrophages, such as, the tumour necrosis factor alpha (TNF-α) (73), interferon-gamma (IFN-γ), and the transforming growth factor-1 (TGF-1) (74, 75). The transcription factors MITF, MAF, THRA, and NR1H3 were proven to bind around a single nucleotide polymorphism (SNP) (rs41505344) in the upstream transcriptional region of MSR1, suggesting an indirect role for the SNP in the transcriptional regulation of MSR1. Moreover, this SNP was associated with altered serum triglycerides and aspartate transaminase levels in non-alcoholic fatty liver disease (NAFLD) (76).

## Regulation of MSR1 by microRNAs

Gene silencing by microRNAs (miRNAs) is another prominent mechanism of regulation, which combines translational repression and mRNA destabilization of target genes (77). Expression levels of MSR1 correlated with the expression of miRNAs that potentially target MSR1 during the differentiation and maturation of bone marrow derived macrophages (BMDMs) (78). Among these miRNAs we found: miR-24, miR-18b, miR-141, miR-150, miR-155, and let-7e. Inflammatory activation by Toll-like receptors (TLRs) leads to the expression of miR-155 in macrophages and dendritic cells (DCs) (79–81). These studies suggest that a wide range of inflammatory mediators can activate miR-155 through mechanisms mediated by both transcription factors: AP-1 and NF-κB. The predominant gene targeted by miR-155 in myeloid cells was the homeobox gene, HOXA9 (82), in DCs was CD274, encoding the inhibitory receptor ligand PD-L1, and in macrophages MSR1 (83). It has also been described that macrophage NFATc3 acts to upregulate miR-204 which in turn depletes levels of MSR1 and protects against atherosclerosis by limiting lipid uptake and subsequent foam cell formation (84).

## Methylation of the MSR1 promoter region and histone modification

Methylation of gene promoters usually leads to transcriptional silencing. Although the effects of MSR1 promoter methylation are not well studied in pathologies associated with MSR1 deficiency, a negative correlation between MSR1 promoter DNA methylation and gene expression in monocytes has been described (85). In a genome-wide analysis of epigenetic and transcriptional variations, MSR1 was studied in terms of gene expression, methylation, and histone variation in human monocytes, neutrophils, and T-cells (86). They described a robust contribution of cis-genetic factors (promoter methylation, H3K4me3, and H3K27ac) to the transcription of MSR1 in immune cells.

In summary, MSR1 expression is frequently altered in cancer, but specifically in cells of the immune system. Decreased expression is more frequently related to DNA deletion, although epigenetic alterations are also observed. However, high levels of MSR1 are found in tumour-associated macrophages (TAMs) as will be discussed at the end of this review

## Cellular signalling pathways of MSR1

**Figure 6:**
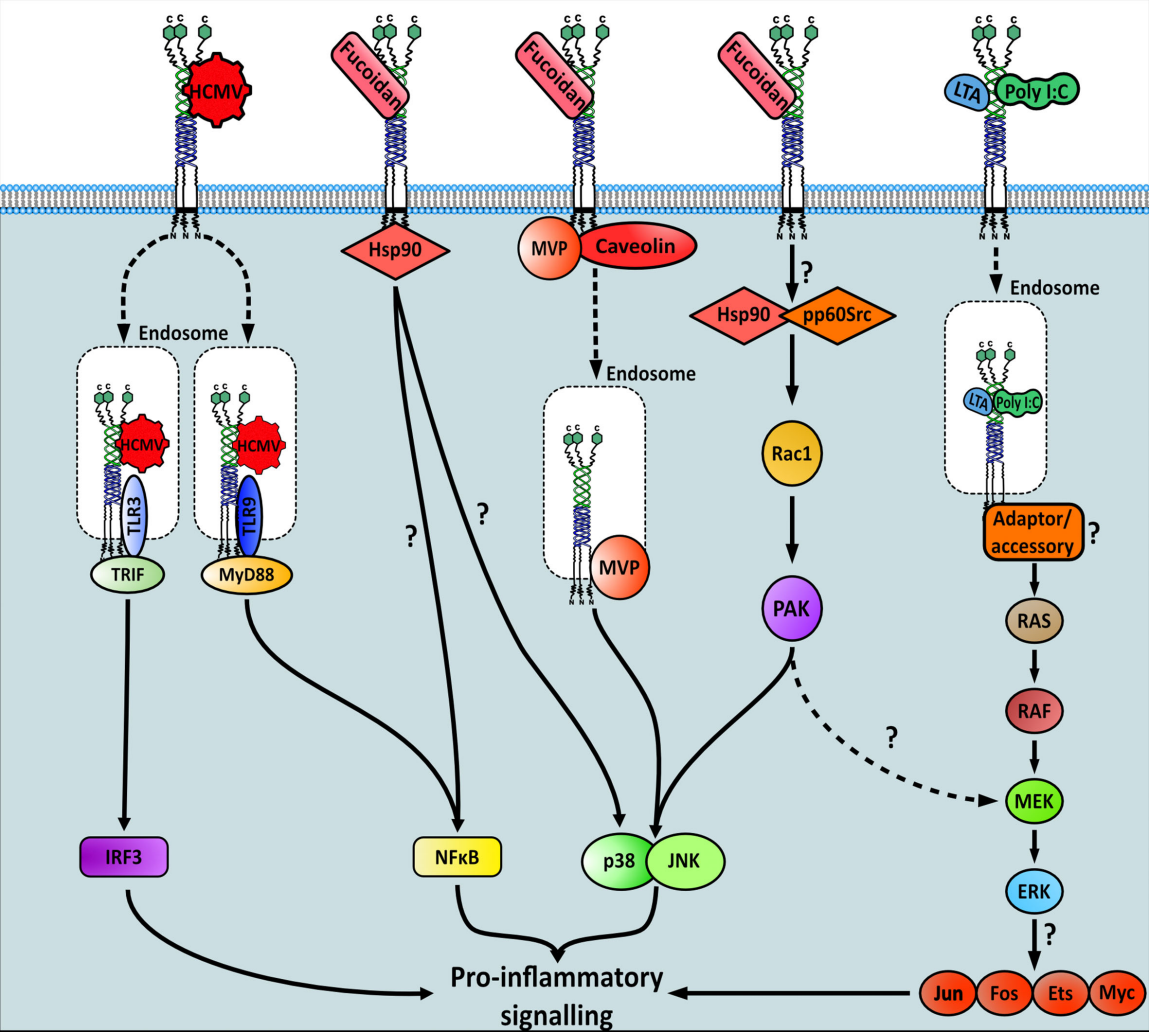
Signalling pathways downstream of MSR1 initiated after specific ligand binding. Question marks within the figure highlight gaps in the knowledge regarding each pathway.

Ligand binding to MSR1 results in receptor internalisation, recruitment of specific binding partners, and the activation of downstream signalling pathways. Which pathway becomes stimulated is both ligand-dependent and macrophage activation state-dependent and whilst a detailed overall pathway surrounding MSR1 is elusive, some signalling cascades have been elucidated, albeit incomplete. Receptor signalling has been most studied in macrophages, these include the fucoidan-induced signalling pathways PTK(Src)/Rac1/PAK/JNK and PTK(Src)/Rac1/PAK/p38 which act to regulate IL-1 secretion (87), and the MAP kinase pathway which induced TNF-α production (88) (**Figure 6**). It is worth noting that neither of these studies could explicitly state how MSR1 activates the resulting pathway. Instead, it was hypothesised that other receptors, heat shock proteins or tyrosine kinases located within the cytoplasm act as binding partners to propagate the signal.

The binding of fucoidan, but not other classical endogenous MSR1 ligands, also stimulated nitric oxide production. This signal was transduced by both p38 mitogen-activated protein kinase (MAPK) and NF-κB-dependent pathways directly downstream of HSP90, which interacted with the cytoplasmic domain of MSR1 (89). The exact cascade involved in either pathway was not interrogated here, therefore it is unclear which proteins are involved post-HSP90 recruitment to MSR1. However, it is possible that the PTK(Src)/Rac1/PAK/p38 pathway is involved (87). Another p38-MAPK mediated signalling response, also stimulated by fucoidan, was found to propagate *via* major vault protein (MVP) binding and the caveolin-mediated endocytic pathway (90). However, as MSR1 has no signalling domains on its short cytoplasmic domain, it remains unclear how binding partners interact. Furthermore, it is not clear how different signalling pathways have been elucidated after binding of the same ligand. Finally, MSR1 bound to human cytomegalovirus (CMV) in monocytes, interacting with toll like receptor (TLR) 3 and 9 on the endosome to stimulate both the IRF3 and NF-κB pro-inflammatory pathways (38).

**Figure 7:**
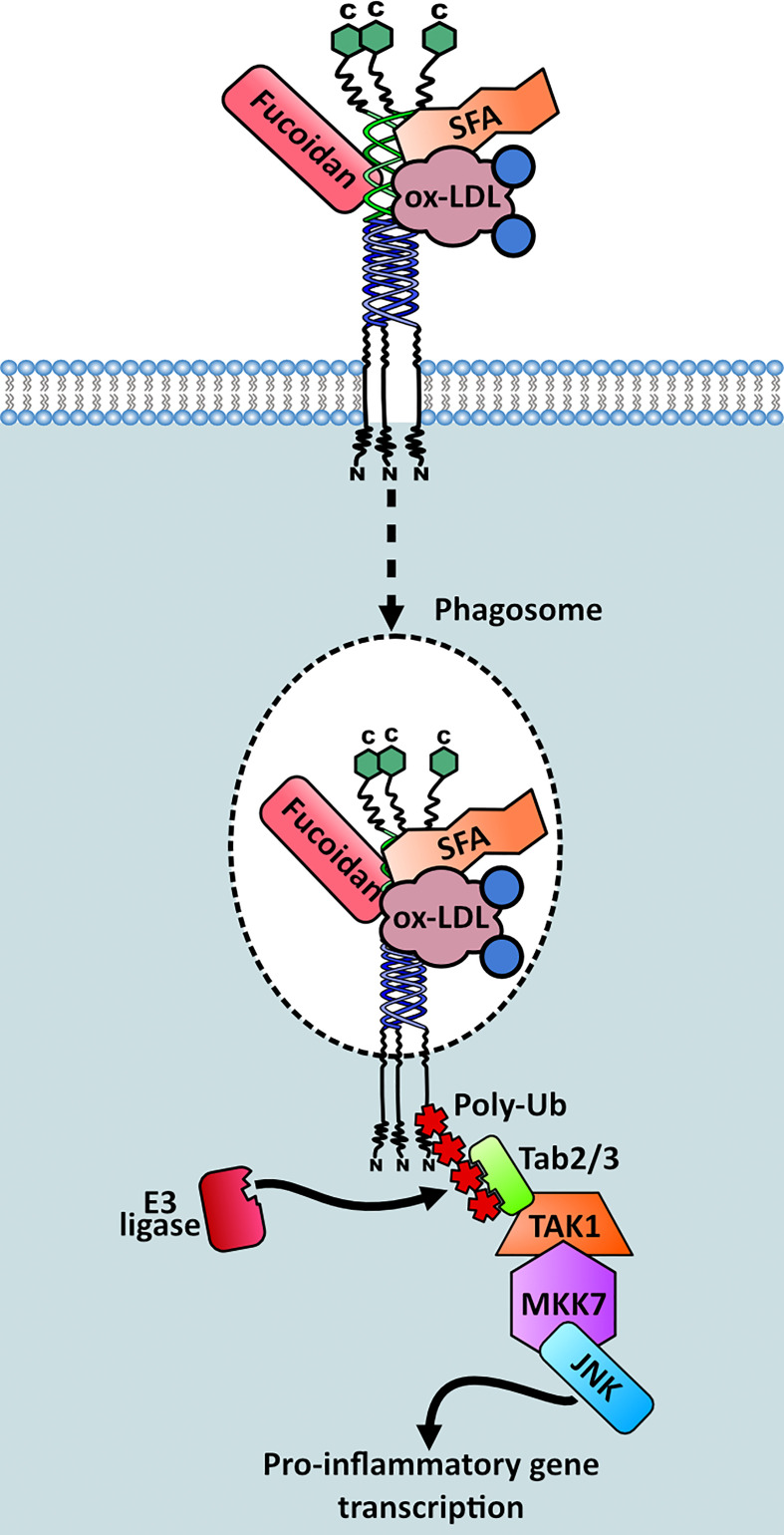
Signalling complex recruitment to MSR1 in M2 macrophages. Triggering of MSR1 by fucoidan, oxidised LDL (ox-LDL) or saturated fatty acids (SFAs) induces MSR1 K63 polyubiquitylation at K27, mediated by an unknown E3 ligase. This polyubiquitin chain acts as a scaffold for recruitment and activation of the TAK1/MKK7/JNK signalling complex. This stimulates a pro-inflammatory phenotypic switch within M2 macrophages.

Our group conducted a study which focused on the dynamic protein changes using proteomics within the phagosomes of M2 macrophages. This unveiled one of the elusive molecular links between MSR1 and its downstream effectors. When triggered by negatively charged polystyrene beads, phagocytosis is induced and MSR1 localises to the phagosome, which acts as a signalling platform providing interaction with innate immune signalling networks. Upon triggering, MSR1 becomes polyubiquitylated at K27 with lysine-63 (K63) chains by an unidentified E3 ubiquitin ligase (**Figure 7**). This polyubiquitylation serves as a scaffold for the recruitment of the TAK1/MKK7/JNK signalling complex (29). Moreover, it has recently been described that MSR1-mediated uptake of saturated fatty acids can also activate JNK signalling and induce Tnf-α and Il-6 expression (76). Triggering of this pathway leads to a phenotype switch from anti-inflammatory to pro-inflammatory. Furthermore, MSR1 K63-polyubiquitin mediated JNK signalling was demonstrated in ovarian cancer patients, indicating a potential role for MSR1 (29). Due to the direct link between MSR1 and the downstream effectors this is one of the most complete signalling pathways surrounding MSR1 elucidated to date (**Figure 7**).

Post-translational modifications (PTMs) play important regulatory roles in biological processes, influencing protein functions, stability, and localisation. However, there is limited information regarding MSR1 regulation by PTMs other than polyubiquitylation as described above. N-glycosylation is one of the most prominent post-translational modifications and is involved in many cellular functions including receptor-ligand interactions, immune response, and pathogenesis of many diseases (91). Two glycoproteomics analyses identified N-glycosylation of MSR1 at N102, N221, and N249 in human diffuse large B-cell lymphoma (92) and in human liver tissue (93). MSR1 glycosylation appears to be relevant to its function since the N102K mutation has been linked to oral squamous cell carcinoma (COSMIC ID: 3715901).

## Clinical implications of MSR1 alterations

**Figure 8:**
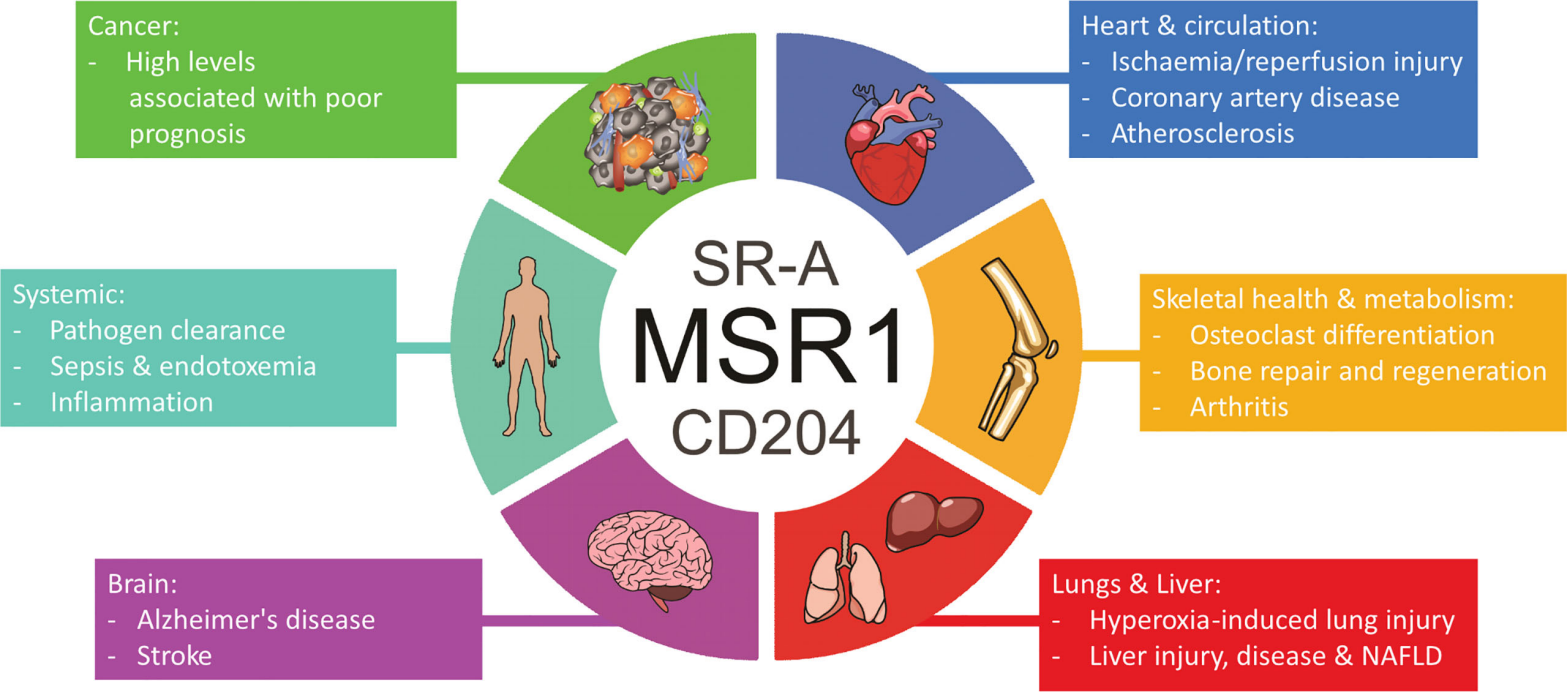
Overview of the involvement of MSR1 in health and disease states.

MSR1 is highly pleiotropic, functioning in various physiological and pathological processes throughout diverse tissues (**Figure 8**). MSR1 acts as a double-edged sword in many processes by either protecting or damaging the body, determined by a plethora of variables, and working through mechanisms many of which remain poorly defined.

## Atherosclerosis

Macrophages in atherosclerotic lesions actively participate in lipoprotein uptake and accumulation, giving rise to foam cells. The most extensively covered role of MSR1 is its activity in atherosclerosis, yet despite the abundance of investigations in this area there is ongoing debate regarding the true role of the receptor. It was first shown to be responsible for the uptake of modified lipoproteins and secretion of inflammatory cytokines leading to atherosclerotic lesion formation (5, 16). However, MSR1 is not the sole receptor responsible for this. Macrophage uptake of modified LDL is mediated through scavenger receptors, mainly CD36 and MSR1, causing accumulation of large amounts of cholesterol within the macrophage. However, MSR1 was found to be more responsible for the binding and degradation of Acetyl-LDL, with CD36 more responsible for oxidised-LDL clearance (94). MSR1 led to greater modified LDL degradation than CD36, indicating that MSR1 may be more implicated in the pathologic breakdown and subsequent cholesterol deposition of modified LDL.

The function of MSR1 in the pathogenesis of atherosclerosis appears to be dependent on the mouse genetic background and apoE presence. However, due to conflicting findings, it is difficult to arrive at a unified hypothesis. *Msr1^-/-^* mice displayed a decrease in atherosclerotic lesion area compared to wild type (95). Similarly, *Msr1^-/-^ ApoE-/-* mice also exhibited a decrease in lesion size (96). Also, in *Msr1^-/-^ Ldlr^-/-^* mice the sizes of atherosclerotic lesions were reduced (19). However, this decrease was less dramatic and would indicate that *ApoE* may be a contributing factor rather than *Ldlr*. For this reason, other scavenger receptors, such as MARCO, CD36, and/or CD68, as well as uptake of very low-density lipoprotein (VLDL) might be involved in foam cell formation during atherogenesis in these mice. In stark contrast to these findings, *ApoE* ^-/-^ *Msr1* ^-/-^ mice fed an atherogenic diet still generated an abundance of foam cells associated with increased aortic lesion area (97). Similarly, *ApoE* ^−/−^ *Cd36* ^−/−^ *Msr1* ^−/−^ mice also showed no decrease in foam cell formation or lesion area. However, a slowed progression towards more advanced necrotic lesions was apparent, accompanied by a decrease in pro-inflammatory gene expression. This study also provides evidence against receptor compensation by CD36 being a cause of variation between investigations (98). Furthermore, mice with a dysfunctional variant of *ApoE*, ApoE3-Leiden, suffered increased susceptibility to atherosclerosis in a similar manner to complete loss of *ApoE*. Interestingly, ablation of *Msr1* also had no significant effect on lesion formation with trends towards more severe lesions apparent (99). Due to disparities in experimental results so far, the role that MSR1 plays in atherosclerosis is still controversial.

Nevertheless, several mechanisms pertaining to the activity and regulation of MSR1 in atherosclerosis have been elucidated in recent years. Perhaps a less obvious protective role played by MSR1 is the uptake of calciprotein particles and mineral debris from the blood circulation, this prevents soft tissue calcification and thus aids in the prevention of calcifying atherosclerosis (23). Intermedin (adrenomedullin-2), a cardiovascular protective peptide often found in atherosclerotic plaques, acts as a negative regulator of *MSR1* expression. Intermedin reduced the uptake and degradation of acetyl-LDL by macrophages, thus decreasing intracellular cholesterol levels. This was due to the increased phosphorylation and decreased ubiquitination of PTEN which acted to stabilise the protein and reduce proteasomal degradation. This in turn diminished *MSR1* mRNA and protein levels (100). Conversely, stimulation with TNF-α or IL-6 increased the accumulation of oxidised-LDL *in vitro* by inducing *MSR1* expression *via* the NF-κB pathway (101). *MSR1* expression can also be induced by angiopoietin-like protein 8 (ANGPTL8), a hormone linked to the regulation of lipid metabolism and the development of atherosclerosis. Overexpression of ANGPTL8 significantly promoted foam cell formation *via* increased cholesterol uptake mediated partially by MSR1 upregulation. However, the exact mechanism behind this upregulation was not determined (102). This highlights again that MSR1 can be attributed to both the pathophysiology of and protection against atherosclerosis.

## Myocardial infarction and ischemia/reperfusion injury

Inflammatory response is an important phase after myocardial infarction and ischemia/reperfusion (I/R) injury. Alternatively activated (M2) macrophages could have enhanced protective effects, they have potential as anti-inflammatory cells, which can reduce immune responses and prevent autoimmune pathology. MSR1, ITGA4, and CYBB have been shown to be up-regulated in both MI and I/R injury (103). Furthermore, MSR1 induced protection by limiting macrophage polarization towards the pro-inflammatory M1 phenotype and therefore decreasing the secretion of IL-1β, IL-6, TNF-α, and MMP9. This aids in the remodelling of the infarct which in turn protects against potentially lethal cardiac rupture. The risk of cardiac rupture with myocardial infarction was increased after targeted knockout of the MSR1 receptor. This resulted in increased activation of the ASK1/p38/NF-κB signalling pathway which mediates apoptosis, thus leading to further myocardial deterioration (104, 105). However, mice lacking MSR1 had a significantly smaller infarct size and better cardiac function following injury in an independent study. This was due to attenuation of p53 mediated apoptosis and, contrary to previous findings, loss of NF-κB signalling. In this scenario, apoptosis was reduced due to increased expression of miR-125b in Msr1-/- macrophages upon myocardial I/R injury and hypoxia/reoxygenation-induced cell damage (106). Whilst the experimental models used here were different in their methodology, they are modelling the same pathophysiologic process, therefore the striking contrast is surprising. The main difference between the two models used is the time spent ischaemic, so perhaps the temporal differences influenced the vastly different signalling consequences observed in Msr1-/- mice.

## Alzheimer’s disease

The microglia, specialised tissue macrophages, of the central nervous system maintain vital processes such as neurogenesis and synaptogenesis, as well as control immune processes in the brain (107). They have been shown to bind and phagocytose β-amyloid fibrils *via* MSR1. This in turn has a rather dichotomous effect. This helps to prevent the formation of the neurotoxic amyloid plaques that contribute towards the development of Alzheimer’s disease. However, parallel to this is the activation of microglia resulting in increased nitric oxide and reactive oxygen species (ROS) production, which can result in damaging neuroinflammation (21, 22). Adding further to the positive effects of MSR1 expression in Alzheimer’s disease, MSR1 expression is reduced in the ageing brain concomitantly with increased β-amyloid deposition and reduced working memory capacity. This results in increased mortality and secretion of proinflammatory molecules (ROS, IL-1β, and TNF-α), and decreased release of anti-inflammatory cytokines (IL-10 and TGF-β) (108). These studies imply that pharmacological enhancement of MSR1 activity or expression may be of potential therapeutic benefit in the treatment or prevention of Alzheimer’s disease. One potential method of enhancing MSR1 activity to aid the clearance of β-amyloid was highlighted during the development of a novel early diagnostic tool for Alzheimer’s disease. Superparamagnetic iron oxide nanoparticles (SPIONs) conjugated to a β-amyloid oligomer specific antibody (W20) and a neuroprotective heptapeptide (XD4) were able to promote the phagocytosis of β-amyloid *via* MSR1 (109). However, apolipoproteins A-I and E have been shown to adhere to MSR1. These apolipoproteins are present in Alzheimer’s plaques and interaction with MSR1 causes macrophage retention, thus leading to increased inflammation in areas already at risk from neurological damage (18). Therefore, therapeutic intervention by the modulation of MSR1 activity should be done with this activity in consideration. However, as microglia from MSR1 knockout mice only showed a 60% decrease in β-amyloid clearance, other scavenger receptors may also be responsible for some of the clearance and signalling changes (110).

## Cerebral reperfusion injury

MSR1 has also been shown to contribute to cerebral reperfusion injury (i.e., stroke) by polarizing macrophages towards the M1 inflammatory phenotype, activity contradicted by later findings in hepatic inflammation (4). This causes inflammatory damage to ischaemic areas and results in a significantly increased infarct size. MSR1 deficiency also attenuated NF-κB activity and apoptotic signalling, both of which normally contribute to ischaemic injury (111, 112). However, contradictory findings have shown that loss of MSR1 leads to reduced clearance of damaged associated molecular patterns (DAMPs), such as HMGB1, more severe inflammation and therefore increased neuronal injury in murine ischaemic stroke (113). This finding was corroborated through the use of a rat model of middle cerebral artery occlusion. Here, enhancement of MSR1-mediated DAMP clearance significantly reduced infarct size and ameliorated neurological deficits. The activity of MSR1 was heightened through the use of a phthalide derivative which induces the MAFB-MSR1 pathway, MAFB being a transcriptional regulator of MSR1 expression (114). This contradiction again mirrors the apparent dualistic nature of MSR1 seen in other disease states, but the use of similar disease models offers no indication as to what drives these differences.

## Skeletal health and bone metabolism

Macrophages have the ability to fuse with other macrophages to form multinucleated giant cells. Commonly viewed as the resident macrophages of bone, osteoclasts are a form of multinucleated giant cell associated with bone repair and regeneration, due to their unique ability to resorb bone (107). MSR1 mediates osteoclast differentiation, potentially by facilitating interaction between osteoclast precursors and osteoblasts, and therefore has a role in normal bone metabolism. Loss of MSR1 decreased osteoclast populations, increasing bone density due to reduced resorption (115). Further to this, involvement of MSR1 has been demonstrated in bone regeneration after fracture. Msr1-/- mice displayed delayed intramembranous ossification, a key step of bone repair where mesenchymal stem cells differentiate into osteoblasts which then deposit mineralized extracellular matrix. This is potentially the result of a reduction in MSR1-mediated osteogenic differentiation of bone marrow stem cells. Moreover, MSR1-mediated promotion of bone repair is controlled through the PI3K/AKT/GSK3β/β-catenin pathway which induces the production of several osteoprotective factors. However, loss of MSR1 inactivates this pathway and subsequently impedes mitochondrial oxidative phosphorylation and M2 polarization (116). Mechanistically, it is unclear exactly how MSR1 modulates this signalling pathway, and therefore requires further investigation.

## Lung injury

Alveolar macrophages serve to phagocytose inhaled particles and respiratory pathogens, therefore MSR1 holds several key protective functions here, owing to its scavenging capabilities. The receptor has been shown to diminish hyperoxia-induced lung injury by limiting macrophage activation thus leading to significantly lower expression of iNOS and slowed generation of TNF-α (117). Alveolar macrophages also limit pulmonary inflammation, in chronic obstructive pulmonary disease and asthma, after oxidant inhalation by utilising MSR1 to scavenge proinflammatory oxidised lipids (118). Furthermore, MSR1 expressed on lung DCs has been shown to control specific immune pulmonary responses to inhaled particles and pathogens by reducing DC migration towards lymph nodes. This therefore mitigates unwanted immune responses to common aeroallergens (119). Not only are MSR1 levels linked to lung injury, but also the MSR1-coding SNP P275A in macrophages was associated with susceptibility to chronic obstructive lung disease in smokers (120).

## Liver injury and disease

Macrophages in the liver, called Kupffer cells, mediate immune response and hepatic tissue remodelling (107). Chronic inflammation or infection in the liver can result in fibrosis, cirrhosis, and hepatocellular carcinoma. Due to the role of MSR1 in lipid uptake, MSR1 deficiency can reduce hepatic inflammation and changes in hepatic lipid metabolism in mice when fed with a high-fat diet (76). However, MSR1 expression is crucial for promoting M2-macrophage activation and polarisation during hepatic inflammation. Expression of MSR1 increases in the later stages of hepatotropic viral infection, shifting the phenotype of non-tissue resident macrophages towards the M2 phenotype. Indeed, the loss of M2-like features was more pronounced in cell lines lacking expression of MSR1 when compared to WT mice. This reveals the importance of MSR1 in the polarisation of macrophages to M2. This phenotype is important in the liver as it is linked with tissue repair which prevents the deposition of fibrotic tissue. MSR1 interacts with MERTK (Tyrosine-protein kinase Mer), MERTK activation may then inhibit the mTOR pathway which acts to modulate macrophage polarization. MSR1 knockdown cells exhibited enhanced mTOR phosphorylation (4). Soluble anti-MSR1 blocked two known key early events during apoptotic cell uptake: the sequential tyrosine phosphorylation of MERTK and of PLCγ2 (20). These data support the MSR1/MERTK complex as a potential target to manipulate apoptotic cell clearance and hence, resolution of inflammation, and infections.

Recent work by our group showed that MSR1 is directly responsible for saturated fatty acids uptake in Kupffer cells and foamy macrophages, leading to an inflammatory response independent of TLR4 in non-alcoholic fatty liver disease (NAFLD). Foamy macrophage generation and fibrosis was also impeded in mice lacking MSR1, indicating potential therapeutic benefit in non-alcoholic fatty liver disease (NAFLD) (76) and other inflammatory diseases, such as atherosclerosis, where foam cells contribute to pathogenesis (121). MSR1 is also upregulated on the cell surface, as well as in its soluble form, in the livers of patients with viral hepatitis infection (122), autoimmune hepatitis disease and in mice subjected to concanavalin A-induced hepatitis (123). Soluble MSR1 binds directly to activated T cells and has an inhibitory effect, potentially *via* an IL-2 dependent mechanism, acting as a negative regulator of CD8+ T-cell activation and expansion. This has a protective effect by limiting hypersensitivity and activation of T-cells. Genetic ablation of MSR1 resulted in a higher sensitivity to concanavalin A-induced liver injury, coinciding with excessive levels of IFN-γ production and STAT1 phosphorylation (123). Homeostatic regulation of T cell activity, mediated by MSR1, becomes especially important in autoimmune hepatitis.

## Response to pathogens

Macrophages are a vital component of the innate immune system and participate heavily in host defence. Therefore, it is no surprise that bacterial and viral components are featured amongst the wide range of ligands that interact with MSR1. MSR1 can bind and clear free bacterial components such as LTA (40) or LPS (39) but can also bind bacteria directly. The receptor has been shown to bind and protect against different gram-positive bacteria: Streptococcus pyogenes (40), S. agalactiae (40), S. pneumoniae (40, 124), Staphylococcus aureus (40, 125), Enterococcus hirae (40), and Listeria monocytogenes (40); as well as gram-negative bacteria: Neisseria meningitides (126), Escherichia coli (127) and Francisella tularensis (128). Whilst mostly host protective, MSR1 has been described to be a negative regulator of multinucleated giant cell formation in tuberculosis. These cells protect the host against Mycobacterium tuberculosis infection by improving antigen presentation and mycobacteria killing (129). MSR1 can differentially regulate bacteria-induced inflammatory response *via* several methods. First by limiting the cell-surface availability of ligands, such as LPS, *via* its scavenging ability. Thus, dampening the TLR4 mediated response. Conversely, the receptor can act as a co-receptor by binding ligands such as trehalose dimycolate, increasing the likelihood of interaction with TLR2. Furthermore, endocytosis by MSR1 enhances the intracellular availability of ligands, allowing interaction with TLRs and nod-like receptors (NLRs) at the endosome (41).

The function of MSR1 in host protection against viruses is varied. The receptor directly binds to, internalises and degrades adenovirus type 5 (130) and protects against herpes simplex virus type-1 (96). MSR1 is also required for the sensing of human cytomegalovirus through interaction with TLR3 and TLR9 (38). Furthermore, it acts as a critical component of TLR3-mediated response to hepatitis C infection by endocytosis of dsRNA (34). The receptor may also mediate autophagy, contributing to the innate response against Chikungunya virus infection (131). Again, the activity of MSR1 in this area is not always protective. The receptor has been shown to contribute towards the pathogenesis of virus-induced fulminant hepatitis by enhancing neutrophil NETosis, a unique form of cell death, and subsequent complement activation (132). Furthermore, MSR1 has been implicated in the pathogenesis of vesicular stomatitis virus (VSV) infection. Mice lacking MSR1 displayed significantly decreased mortality and morbidity, with MSR1 being shown to have a significant impact on VSV infection in the central nervous system. Cellular entry of VSV was dependent on MSR1 functioning as a co-receptor for low density lipoprotein receptor (LDLR) mediated uptake (133).

Recent evidence has emerged indicating a potential role for MSR1 in SARS-CoV-2 infection, however the limited data accrued so far is already contradictory. COVID-19 patients with severe symptoms were found to have significantly increased MSR1 expression on circulating monocytes and DCs (134). M2 macrophages infected with SARS-CoV-2 also showed increased MSR1 expression after 48 hours (135). However, in a separate study, the spike protein of SARS-CoV-2 was shown to reduce the expression of MSR1 on macrophages *via* interaction with DDX5, a protein involved in pre-mRNA splicing (136). Blood transcriptome data gathered from patients with severe COVID also showed decreased MSR1 expression (137). Ultimately, the regulation of MSR1 expression and resulting signalling consequences in SARS-CoV-2 infection are currently unknown.

The molecular mechanism behind MSR1 mediated TLR4 signalling inhibition was the first endocytosis and phagocytosis independent MSR1 signalling mechanism to be described. This mechanism acts as a fine control for LPS-induced TLR4-NF-κB signalling. MSR1 regulates this pathway by directly interacting with the TRAF-C domain of TRAF6, this prevents its dimerization or ubiquitylation which is normally required for TLR4-mediated activation. This prevents TRAF6 from activating IκB kinase and the MAPK cascade. The resulting sequestration of NF-κB prevents the activation of target genes involved in immunity and inflammation and ultimately dampens the adaptive immune response driven by TLR4 activation (138).

## Endotoxemia and sepsis

Further to its role in bacterial immune response, MSR1 has a complex and contradictory role in endotoxemia or systemic inflammation, often used as a model substitute for sepsis despite clear disparities and limitations in their nature (139). Several studies indicate that MSR1 ameliorates sepsis by suppressing the pro-inflammatory response, in particular by reducing TNF-α signalling and TLR4-induced activation of NF-κB, potentially by limiting the availability of free LPS (140–144). Further to this, selective and transient depletion of Msr1 in macrophages *in vivo* resulted in elevated serum concentrations of TNF-α and IL-6 as well as decreased survival rate when mice were challenged with LPS-induced endotoxemia (145). On the contrary, MSR1 has been shown to enhance the production of TNF-α in LPS-treated J774A.1 macrophages (146). This finding was recapitulated in a separate study, however the inclusion of fucoidan alongside LPS treatment may account for the increased pro-inflammatory signalling observed (29, 147). Further contradicting the protective nature of MSR1, LPS treatment resulted in a higher mortality in wild type mice than in Msr1-/- (148). MSR1 played a clear detrimental role in the disease pathophysiology of the cecal ligation and puncture model of sepsis, enhancing pro-inflammatory signalling through interaction with TLR (149, 150). Further to this, blockade of MSR1 by berberine alkaloids restricted the receptors’ ability to endocytose LPS. This prevented downstream activation of the caspase-11 pathway which normally acts to induce endotoxin-mediated coagulation syndrome, often seen in bacterial sepsis (31).

The contradiction in findings here, as in atherosclerosis, offers no concrete understanding of the molecular mechanisms. Moreover, the inclusion of fucoidan when investigating pro-inflammatory response may confound results as it has since been shown to induce JNK signalling. Furthermore, differences in expression or structure of MSR1 may, as evidenced between C57BL/6J and A/J mice, lead to differences in inflammatory response (142). LPS is known to activate TLR4 and induce the expression of pro-inflammatory cytokines through the MAPK kinase and NF-kB pathway (151). Therefore, the controversial role of MSR1 in sepsis could be masked by the activation of other receptors by LPS.

## Rheumatoid arthritis and osteoarthritis

MSR1 has been investigated for its use as a biomarker and the pathological role it plays in rheumatoid arthritis (RA), a highly debilitating chronic autoimmune disease. Early detection is dependent on sensitive and specific biomarkers. At current the two clinically used biomarkers, rheumatoid factor and anti-cyclic citrullinated peptide antibody (anti-CCP), only show moderate discriminatory ability. A large-scale multicentre study revealed that soluble MSR1 could be utilised as a diagnostic marker for RA, offering competitive sensitivity and specificity values. Further to its diagnostic value, the role of MSR1 in RA pathogenesis was correlated with disease severity in two different models: 1) the administration of soluble MSR1 which increased disease severity, and 2) inhibition or genetic ablation of MSR1 which reduced disease severity. MSR1 mediated disease progression was concomitant with increased T cell activation. Resistance to RA in Msr1-/- mice was associated with decreased IL-17a and TNF-α production by T helper cells (152).

In osteoarthritis (OA), macrophages exacerbate cartilage damage through the production of pro-inflammatory cytokines. Macrophage activation is a key step in OA initiation along with upregulation of other scavenger receptors, and the release of polyanionic molecules. Utilising dextran sulphate nanoparticles to encompass triamcinolone acetonide, a corticosteroid used in OA treatment, MSR1 was directly targeted to reduce the viability of macrophages. This in turn diminished pro-inflammatory cytokine mediated cartilage damage (153). Similarly, MSR1 can be targeted for the treatment of RA using methotrexate in place of triamcinolone acetonide (154, 155). These studies show that, whilst MSR1 is upregulated and implicated across the spectrum of disease, the ligand binding and endocytic capabilities of the receptor can be exploited to deliver disease modifying drugs specifically to macrophages.

## MSR1-positive tumour associated macrophages in cancer

**Figure 9:**
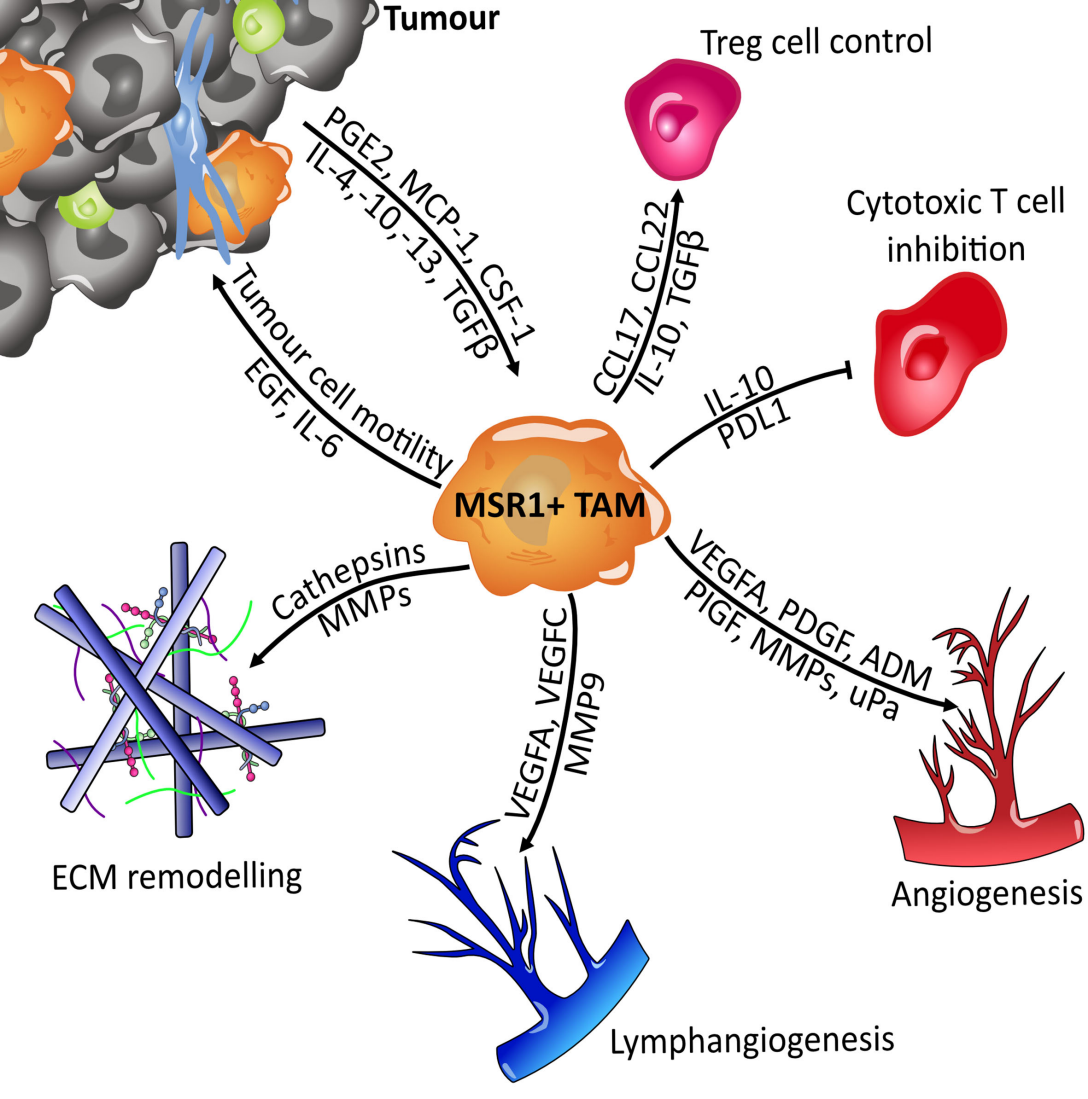
Overview of MSR1-positive TAMs signalling. Macrophages become polarised towards the M2-like tumour-associated macrophage (TAM) phenotype by tumour derived factors such as IL-4 and CSF-1. Once polarised they then support tumour expansion and metastasis by secreting factors that influence several of the hallmarks of cancer. They suppress the immune system by regulating the function of T_reg_ (Regulatory T cells) and cytotoxic T cells (CD8+). Metastasis and growth are facilitated through breakdown of the extracellular matrix (ECM), the induction of angiogenesis and lymphangiogenesis, and directly through cytokine mediated influence of tumour cell motility. Adrenomedullin (ADM); colony stimulating factor 1 (CSF-1); epidermal growth factor (EGF); interleukin (IL); matrix metalloproteinases (MMPs); monocyte chemoattractant protein-1 (MCP-1); placental growth factor (PlGF); platelet-derived growth factor (PDGF); programmed death-ligand 1 (PDL1); prostaglandin E1 (PGE-1); transforming growth factor beta (TGFb); urokinase plasminogen activator (uPa); vascular endothelial growth factor A (VEGFA).

Tumour associated macrophages (TAMs) can be held partially responsible for the propagation of tumours through their action on several of the hallmarks of cancer: avoidance of immune destruction, activating invasion and metastasis, or even in angiogenesis (**Figure 9**) (156–160). Macrophage phenotype becomes shifted towards the immunosuppressive M2-like subset by tumour-derived cytokines such as IL-4, IL-10, and IL-13. TAMs are able to inhibit the action of cytotoxic T-cells *via* IL-10 secretion and support regulatory T-cells, thus leading to immune evasion and tumour proliferation (157). Following this, TAMs secrete pro-migratory factors such as epidermal growth factor (EGF), cysteine cathepsins and matrix metalloproteinases (MMPs). TAMs located at the migratory front of the tumour utilise these matrix-degrading enzymes to aid migration by breaking down the extracellular matrix (ECM) (161, 162). Once migration has been facilitated, the next barrier in benign-to-malignant tumour transition is hypoxia caused by a deficiency in vasculature. Therefore, an angiogenic switch is required to allow neovascularisation and the supply of oxygen to the growing tumour. This switch is provided by TAMs with the secretion of angiogenic factors such as VEGF-A, VEGF-C, and adrenomedullin. Pro-angiogenic cytokines such as uPA are also produced. The MMPs produced by TAMs also have a secondary effect, aiding angiogenesis by allowing the release of extra growth factors from extracellular depots (157, 163). Lymphangiogenesis is also stimulated in a similar fashion to angiogenesis, this further increases the migratory potential of the tumour. It is therefore clear that TAMs have an important role to play in tumorigenesis.

MSR1 is emerging as an important TAM marker, with recent studies linking high expression levels of the receptor to a significantly poor prognosis and increased severity of multiple forms of cancer. However, the exact mechanistic role that MSR1 plays in TAM-mediated tumorigenesis remains elusive.

Whilst deletion of MSR1 and the surrounding chromosome region in tumour cells has been associated with poor cancer prognosis (**Figure 4**), the expression of MSR1 in tumour cells is ultimately low. Therefore, the correlation between MSR1 expression in tumour cells and carcinogenesis has not been well studied so far. However, there are an abundance of studies that show that MSR1 is an essential marker for TAMs and is associated with cancer progression and poor prognosis. However, the use of one single marker may seem like a reductionist approach, therefore multiplexed immunohistochemistry (IHC) has been developed to allow delineation of specific macrophage subpopulations within the tumour microenvironment (TME). Several sub-populations can be identified using M1 markers (HLA-DR/CD68), M2 markers (CD163/CD68), pan-macrophage markers (CD68/CK), and MSR1 as a TAM marker (164). It has recently been published that not only is the MSR1 marker relevant in tumour prognosis but also the ratio of T cells, B cells, and MSR1+ TAMs. A high ratio of CD8+ T cells to MSR1+ TAMs indicated a favourable postoperative prognosis in prostate cancer (165). Similar prognostic value was confirmed in thymic carcinoma where the ratio of CD8+ T cells/MSR1+ TAMs and CD20+ B cells/MSR1+ TAMs indicated prognostic effect in the stroma (166).

MSR1+ TAM populations have the potential to be used as an effective prognostic marker for various cancers. A higher number of MSR1+ TAMs were present in the tumour stroma area than in the primary tumour and this was associated with multiple clinicopathological factors, poor prognosis, and shorter survival time in non-small cell lung cancer (NSCLC) (167), lung squamous cell carcinoma (168), lung adenocarcinoma (169, 170), uterine cervical adenocarcinoma (171), invasive ductal carcinoma (172), glioma (173), and muscle-invasive bladder cancer (174). Moreover, MSR1+ TAMs present more levels of IL-10 and MCP-1 which are involved in accumulation, migration, and polarisation of M2 macrophages (168, 169). In addition, MSR1 levels in TAMs are shown to have a positive correlation with the cancer’s grade in glioma (175) and colorectal adenoma (176). Furthermore, highly proliferative cells can induce macrophage colony-stimulating factor (M-CSF) which increases MSR1 expression and M2 polarisation in macrophages (175, 177).

TAMs have been associated with the promotion of tumour metastasis. Besides the use of IHC to analyse MSR1+ TAMs, flow cytometry also provides a multiparameter analysis to study cell invasion and tumour metastasis of breast cancer (178), gastric cancer (179), oral squamous cell carcinoma (180), and ovarian cancer (181). The highly complex and immune cell-rich desmoplastic stroma contributes significantly to tumour cell migration and metastasis. Many studies have indicated that tumour cells can promote M2 polarisation of TAMs inducing the upregulation of chemokines, cytokines, and matrix metalloproteinases associated with tumour promotion, such as: TNF-α, MMP-1,-2,-7,-9,-14, VEGF-B,-C, and CSF-1 (179, 182, 183). In pancreatic cancer, MSR1+ macrophages were found within the TME and at the migratory front of the cancer which was associated with tumour aggressiveness (184). Comparison studies of brain metastases and primary lung tumours have indicated an increased number of MSR1+ TAMs in the TME of these metastases, but not from other cells of the immune system (185). Furthermore, infiltration of MSR1-positive macrophages into the TME of colorectal cancer was connected to poor prognosis due to increased proliferation and invasion of tumour cells (186). A significant link between the number of MSR1+ macrophages and lymphangiogenesis was also observed in pancreatic ductal adenocarcinoma thus providing insight into how these tumour-promoting macrophages facilitate metastasis (184). However, this association has been contradicted by recent weighted gene co-expression network analysis (WGCNA) of immune infiltration cells in osteosarcoma metastasis. This study highlighted that macrophage infiltration was decreased in these metastases and MSR1 was identified as having an anti-metastatic role and linked to an increase in overall survival (187). Although, as this analysis was carried out using transcriptome data, these findings need experimentally corroborating through the use of other omics methods.

Whilst the relationship between MSR1 and cancer has mainly been represented as a correlation between increased expression and poor prognosis, there is also evidence that indicates isoform driven effects in cancer. Isoforms of MSR1 were differentially expressed in primary melanomas and benign melanocytic nevi. One isoform, more prevalent in melanoma, was characterised by a gain of collagen and SRCR domains. As the collagen-like domain is the site for ligand binding, this may indicate that cancer derived ligands work *via* MSR1 to help drive the formation of the metastatic form of the disease. On the other hand, the most upregulated isoform in benign nevi was a non-coding transcript with loss of an open reading frame (188). Further suggesting a role for MSR1 in benign to malignant transformation of tumours. The clinical studies outlined above all relied on IHC analysis to investigate the potential for MSR1 positive TAMs to be used as a prognostic marker in several tumours. Whilst these investigations provide valuable insight into the link between these TAMs and aggressiveness of the tumour, they offer limited functional or mechanistic insight into the pathophysiologic activity of MSR1 in the TME. Mechanistic insight into this relationship was explored by culturing human monocyte-derived macrophages with conditioned media from three different breast cancer cell lines. Conditioned media was shown to stimulate MSR1 over-expression but not CD163 or CD206, two typical markers for M2 macrophages. Thus, showing that the increased expression of MSR1 is a result of a more specific reprogramming rather than a simple M2-like switch (172). Proteomic analyses have attempted to discover the tumour cell ligands that could activate MSR1 in TAMs (189). Unfortunately, the cancer cell derived molecules responsible for MSR1 upregulation are yet to be identified. A potential candidate, however, is hyaluronan expression of which correlates highly with the number of M2 macrophages in the TME of malignant breast tissues. Furthermore, inhibition of hyaluronan synthesis or its receptor, CD44, significantly reduced M2 polarisation and MSR1 expression (190). This gives one possible explanation as to how MSR1 becomes significantly upregulated in various cancers. More interestingly, hyaluronan is an anionic biopolymer, therefore it might act on and signal through MSR1 directly within the TME (191). This is yet to be demonstrated experimentally.

Taken together, there is striking evidence that MSR1 is a valuable prognostic marker for the severity of multiple types of cancer. Furthermore, there is mounting evidence that MSR1 itself holds some pro-tumour activity with targetable signalling pathways remaining elusive. However, the high expression of MSR1 in the TME, coupled with its ligand binding capabilities, make it a promising candidate for pharmacologic intervention.

## Therapeutic strategies

The first approach was to use the MSR1 ligands themselves, which can potentially competitively inhibit its function. Fucoidan, poly I, and poly G were able to inhibit tumour progression and invasion *via* MSR1 in ovarian and pancreatic cancer models (182). However, the caveat to consider here is that ligands will not act solely as inhibitors, as different ligands will often activate different inflammatory signalling pathways. Further therapeutic potential was identified by exploiting the ligand binding capabilities of MSR1 to generate a highly selective conjugate for TAM-targeted photodynamic therapy (PDT). Polyanionic sodium alginate was conjugated to phthalocyanine, a clinical photosensitiser, to target MSR1 and accumulate in TAM-rich areas. The sodium alginate-phthalocyanine conjugate achieved an 87% tumour inhibition growth rate. Coupled with low toxicity to normal tissue, these results show promise in targeting TAMs *via* MSR1 to reduce their effect on tumour growth and metastasis (192). It is clear that MSR1 acts within the TME, however a defined tumour-specific ligand and signalling cascade are yet to be clarified, this therefore requires further investigation.

To date, no specific inhibitors for the MSR1 receptor have been described. However, a small 16-amino acid amphipathic peptide, 4F, inhibited MSR1 and drastically reduced the invasion of ovarian and pancreatic cancer cells, and reduced tumour growth *in vivo* (189). Furthermore, tumours with MSR1 mutations increased sensitivity to treatment with the AKT inhibitor GSK690693, according to the Genomics of Drug Sensitivity in Cancer (GDSC) project using the Pan-cancer database (193).

Adding further complexity to the effects of MSR1 in cancer, upregulation of the receptor is not always associated with poor prognosis. Irradiated mice which received transplanted bone marrow from Msr1−/− mice developed chronic myeloid leukaemia (CML) significantly faster than those which received WT cells. Furthermore, ectopic overexpression of MSR1 delayed CML development. The tumour promoting effect of loss of MSR1 was determined to be a result of activation of the PI3K-AKT-GSK3β pathway and increased expression of β-Catenin. This indicates that enhancement of MSR1 function may be a novel therapeutic intervention for CML (194).

Vascular leukocytes (VLCs) are similar to TAMs as they are recruited to the tumour and phenotypically switch to a tumour-promoting population. MSR1 was also found to be expressed in the VLCs in ovarian cancer. Therefore, an antibody-based method was developed to deplete VLCs from the peritoneum, using saporin toxin (ZAP) conjugated to the 2F8 anti–SR-A monoclonal antibody. This immunotoxin was able to specifically deplete the VLC population and reduce tumour burden (195). In another context of disease, therapeutic inhibition of MSR1, *via* monoclonal antibody treatment, decreased the release of TNF-α both in an NAFLD mouse model and in ex vivo human liver (76).

MSR1 expression significantly correlated with immune checkpoints (173). Suggesting that MSR1 positive TAMs synergise with immune checkpoint regulators to inhibit the activity of T cells in the TME. This can promote tumour progression and limit the efficacy of therapeutic interventions designed to prime the adaptive immune system. MSR1 is known to bind and internalise multiple heat shock proteins (HSP) such as HSP27 (25), HSP110 and GRP170 (26), and GP96 and GRP94 (27). Interaction of MSR1 with HSPs subsequently induces an immunosuppressive response by dampening TLR4, NFkB and MAP kinase activity (196). The lack of MSR1 significantly enhanced HSP- or LPS-mediated vaccine activity against poorly immunogenic tumours (197, 198). HSP-adjuvant vaccines function on the basis that TLR4 signalling can be targeted to lead to the stimulation of a heightened immune response against tumour-associated antigens. Therefore, the finding that loss of MSR1 further enhances this immune response indicates cross talk between MSR1 and TLR4 signalling. This increase in antitumour response could be attributed in part to an enhanced CD8+ T cell response (197). MSR1 may act to hinder the efficacy of therapeutics designed to exploit the adaptive immune system. It was further demonstrated that MSR1 inhibition or deletion improved the ability of DCs to generate antitumour responses to melanoma, improving the expansion and activation of CD8+ T cells specific for melanoma antigens. Adding further depth to these findings, the use of Msr1-/- DCs to produce antigen-targeted vaccines resulted in increased infiltration of not only CD8+ T cells but also natural killer (NK) cells as well as increased intratumoural ratios of both CD4+ and CD8+ T effector cells to CD4+CD25+ T regulatory cells, all of which contribute to the elimination of malignant cells (199). Furthermore, MSR1 has been shown to impair the cytotoxic antitumour response of NK cells post-surgery. This impairment was linked to an increased expression of MSR1 on NK cells following surgery, resulting in increased lipid accumulation. Lipid-laden NK cells subsequently showed a decrease in expression of important NK cell receptors and decrease in the ability to lyse tumours (200).

The immunomodulatory effects of MSR1 were also confirmed during radiation therapy for prostate cancer. Combination of radio and immunotherapy is beneficial in local tumour control as irradiation results in tumour-specific antigen shedding. These antigens can then be processed by antigen presenting cells such as DCs, ultimately resulting in an anti-tumour immune response. *In situ* vaccination with DCs in which MSR1 had been downregulated, alongside ionizing radiation, significantly suppressed the growth of murine prostate cancer and a reduction in distant metastases was also seen. Recapitulating earlier findings, a significant increase in tumour infiltrating CD8+ T cells was identified (201).

## Discussion

**Figure 10:**
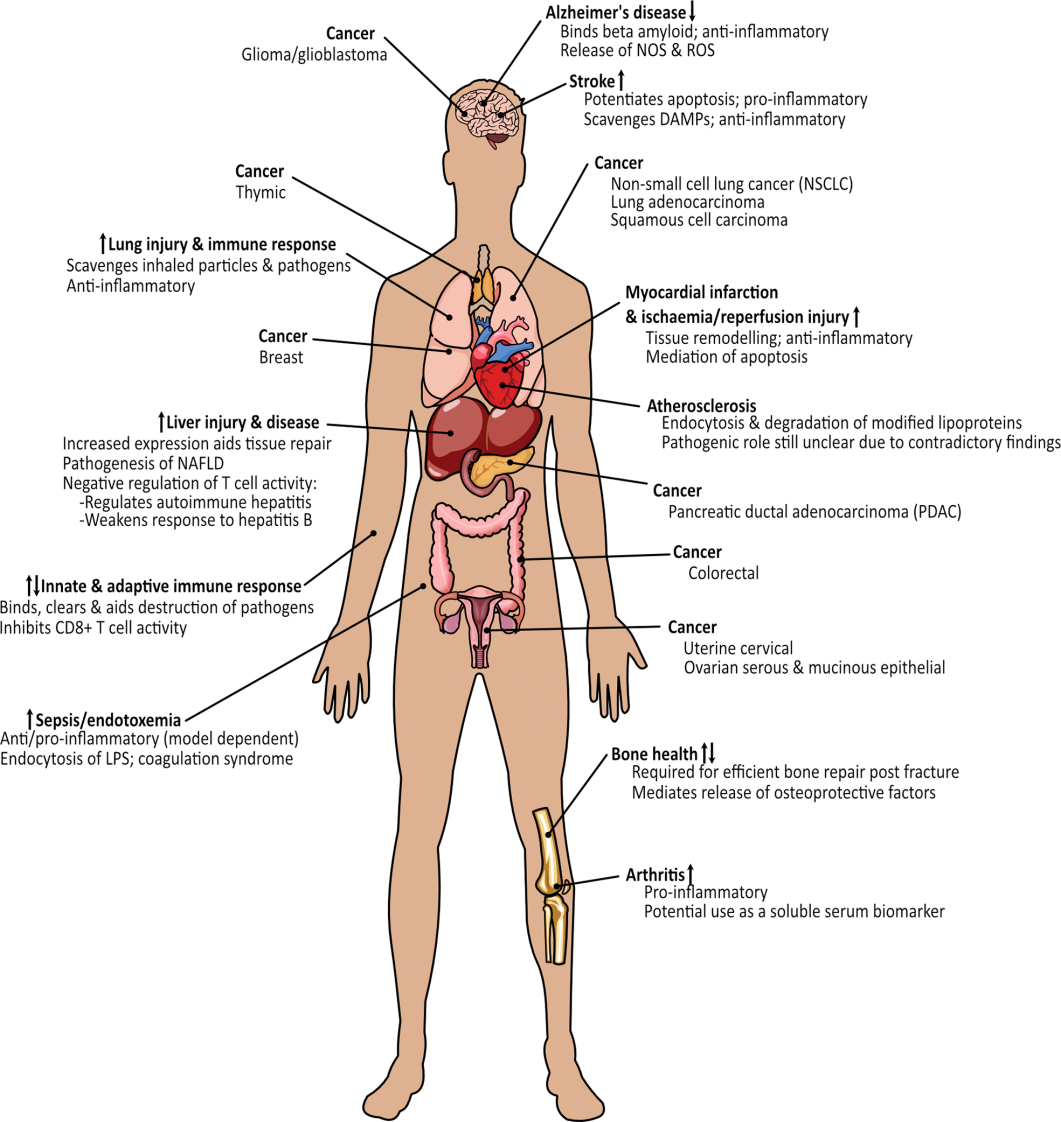
Overview of the processes and diseases where MSR1 holds either a protective or damaging function. Arrows indicate whether expression of MSR1 is increased or decreased.

In this review, we have explored the physiological and pathophysiological importance of MSR1 in different tissues (**Figure 10**), and more specifically, the changes influenced by post-translation modifications, differential expression, mutation, or ligand binding, which may have consequences for MSR1 activity and its function in macrophages. MSR1 holds canonical physiological roles mediating endocytosis of modified lipids, phagocytosis of pathogens and apoptotic cells, cell adhesion, and cytokine production. However, more recently, MSR1 has been implicated in various signalosomes that trigger inflammatory and tumorigenic pathways. The ability of MSR1 to either ameliorate or potentiate disease is, in part, a result of the wide ligand binding capabilities of MSR1, giving rise to multiple different signalling pathways. Contradictory findings of MSR1 activity in diseases such as atherosclerosis and sepsis may also potentially indicate a much more complex mechanism behind the activity of MSR1. The intrigue here, however, stems from the fact that MSR1 holds no distinct intracellular signalling motif. This leads to one of the widest gaps in the knowledge surrounding the signalling of MSR1, with only one full signalling pathway identified so far. This being ubiquitin chain-mediated recruitment of the TAK1 complex on the phagosome. Identification of binding partners, such as HSP90, or post-translational modifications, such as ubiquitin chain linkages or phosphorylation, that facilitate MSR1 signalling changes will bridge this gap and offer a greater insight into how exactly MSR1 can influence such a range of processes.

Furthermore, the signalling pathways outlined thus far all have pro-inflammatory consequences. This is interesting as MSR1 is most often correlated with the anti-inflammatory M2 macrophage phenotype, especially in the TME. Therefore, investigations into anti-inflammatory signalling downstream of MSR1 are vital to fully understand the influence of MSR1 expression in inflammatory disease. Manipulating M2 macrophages through MSR1 may represent a new targeted therapeutic approach for diseases such as cancer, arthritis, and other inflammatory diseases. Proving that targeting cells that promote disease progression, rather than the disease itself, is a viable therapeutic option and that MSR1 is an ideal receptor to do so due to its wide ligand-binding capabilities and endocytic function. However, one caveat that is often overlooked in MSR1 analyses is the use of fucoidan, or other MSR1 ligands, as inhibitors. As is clear from the pro-inflammatory pathways previously described, fucoidan does not act as an inhibitor of MSR1, in fact it is very much the opposite, activating multiple different pathways. Therefore, studies which utilise fucoidan to block the activity of MSR1, or other ligand binding to MSR1, must take this into account when interpreting results as fucoidan may partly re-polarise M2 macrophages towards an inflammatory phenotype. The use of ligands as inhibitors may account for some of the differences seen between experimental models. Differences may also be seen due to the plastic nature of macrophages influencing the availability of signalling molecules, co-receptors, or post-translational machinery, as it is clear that MSR1 relies on these to stimulate signalling cascades.

Overall, it is evident that MSR1 plays a dual role in a multitude of difference processes. However, there is still a long way to go before the receptor is fully understood. A full understanding may be accomplished through deeper interrogation of MSR1 signalling. This is especially important to understand its role in cancer, where detailed molecular mechanisms are absent despite a wealth of data indicating its role as a prognostic marker for disease severity. Further to its activity on macrophages, the influence of MSR1 on other adaptive immune cells such as B cells, T cells and NK cells is also largely unknown. Determining how MSR1 communicates with other cell types may also reveal how MSR1 influences such a wide variety of processes. Pharmacologic modulation of MSR1 activity in disease may also better indicate the true role of the receptor. Further insights in the future will likely shed light on the molecular details of MSR1 functions, thus clarifying its clinical value for each inflammatory pathology and cancer.

## Author contributions

JG planned the concept and design of the review. JG and JM-R collected previous literature on the topic and drafted the article. JG made the figures. MT performed critical revision of the article. JM-R and MT edited the final version of the article. All authors contributed to the article and approved the submitted version.

## Funding

This work was partly funded by a Welcome Trust Investigator Award (215542/Z/19/Z) to MT. JG was funded by a MRC Studentship of the MRC DiMeN Doctoral Training Partnership.

## Acknowledgments

The Genotype-Tissue Expression (GTEx) Project was supported by the Common Fund of the Office of the Director of the National Institutes of Health, and by NCI, NHGRI, NHLBI, NIDA, NIMH, and NINDS. The data used for the analyses described in this manuscript were obtained from the GTEx Portal on 20/07/22. The results published here are in part based upon data generated by the TCGA Research Network: https://www.cancer.gov/tcga.

## Conflict of interest

The authors declare that the research was conducted in the absence of any commercial or financial relationships that could be construed as a potential conflict of interest.

## Publisher’s note

All claims expressed in this article are solely those of the authors and do not necessarily represent those of their affiliated organizations, or those of the publisher, the editors and the reviewers. Any product that may be evaluated in this article, or claim that may be made by its manufacturer, is not guaranteed or endorsed by the publisher.
